# The implementation of a new measles vaccine mandate in Germany: A qualitative study in local health departments

**DOI:** 10.1371/journal.pone.0306003

**Published:** 2024-06-25

**Authors:** Sophia Werdin, Julia Neufeind

**Affiliations:** 1 Swiss Tropical and Public Health Institute, Swiss Centre for International Health, Allschwil, Switzerland; 2 University of Basel, Basel, Switzerland; 3 Immunisation Unit, Robert Koch Institute, Berlin, Germany; Shahrood University of Medical Sciences, ISLAMIC REPUBLIC OF IRAN

## Abstract

**Background:**

Measles is a highly contagious disease with the potential for severe complications. Despite the availability of effective vaccines, there have been recurrent measles outbreaks in Germany over the past decades. In response, a new measles vaccine mandate was introduced on March 1, 2020, aimed at closing vaccination gaps in high-risk populations. This study evaluates the mandate’s implementation, identifies operational challenges, assesses the impact of the Coronavirus disease 2019 pandemic, and investigates expert attitudes towards the new policy.

**Methods:**

Semi-structured expert interviews were conducted with staff members of 16 different local health departments in Germany. The interviews, carried out in April and May 2021, were electronically recorded, transcribed verbatim, and analyzed using the Framework method.

**Results:**

The implementation of the measles vaccine mandate in local health departments varied substantially. Challenges in implementing the mandate primarily arose from uncertainties regarding procedural specifics, such as handling fraudulent medical certificates and imposing sanctions, leading to a call from many interviewees for uniform guidelines to ensure coherent implementation. At the time the measles vaccine mandate came into force, managing the Coronavirus disease 2019 pandemic was a priority in most local health departments, often delaying the implementation of the mandate. Despite the difficulties encountered, most experts considered the mandate to be an effective step towards measles elimination.

**Conclusions:**

The measles vaccine mandate has imposed a new responsibility on staff in German local health departments, which is associated with implementation challenges such as procedural uncertainties and vaccine hesitancy, but also the Coronavirus disease 2019 pandemic as a contextual impediment. Significant differences in the implementation approach underscore the need for harmonization to enhance implementation efficiency and public acceptance of the mandate. Despite the mandate’s potential to increase vaccination rates, our findings advocate for a comprehensive approach, incorporating public education, accessible vaccination, and measures to address social disparities.

## Introduction

The new measles vaccine mandate in Germany is intended to increase measles immunity in the population and to help eliminate measles [1,2]. Measles is one of the most contagious infectious diseases in humans and can cause complications such as pneumonia and potentially fatal diseases such as panencephalitis [3,4]. The most effective preventive measure is vaccination [4]. Although safe and effective vaccines have been available for many years, measles outbreaks continue to occur worldwide [5,6]. Since 1984, measles elimination has been a declared goal of the member states of the World Health Organization’s European Region [7]. This goal can only be achieved when at least 95% of the population is immune to measles [3,4,8]. In Germany, there has been no considerable progress in increasing vaccination rates in recent years. Vaccinations are often administered too late, especially the second dose, and there are vaccination gaps in all age groups [8]. Nationwide, in the 2017 birth cohort, 83.5% of children have received the first dose by 15 months of age, as recommended by the National Immunization Technical Advisory Group in Germany (STIKO) [9]. 69.9% of children in the 2016 birth cohort received their second dose in accordance with STIKO recommendations by 24 months of age [9].

These circumstances motivated the introduction of a new measles vaccine mandate in Germany in March 2020 [2]. This mandate requires specific groups of people in certain facilities, such as children in preschool childcare and schools as well as staff in health care facilities and individuals in community shelters, to provide proof of measles immunity [10]. Those who intend to enter a relevant facility after March 2020 must immediately provide proof [10]. For individuals already working or being cared for in relevant facilities before March 2020, a transition period was set until July 2021. Due to the Coronavirus disease 2019 (COVID-19) pandemic, this period was later extended to July 2022 [11].

The vaccination requirements are based on the recommendations of the STIKO and must be proven with the vaccination card or a medical certificate from a physician [10]. A proven medical contraindication is the only reason for exclusion from the regulations [10]. In addition, the mandate also regulates the responsibilities of different actors in its implementation, including local health departments (LHDs), which play a key role in this context. For example, LHDs are commissioned to follow up on the missing proofs of immunity, to carry out vaccination consultations, and to impose sanctions if the immunity proof is not provided [10]. These sanctions include penalties of up to EUR 2,500 and restricting access to relevant facilities [10]. Accordingly, the mandate has brought new responsibilities to LHDs.

Since spring 2020, LHDs in Germany have been engaged in efforts related to the COVID-19 pandemic response, which has resulted in considerable additional workload, pushing some LHDs to their operational capacity limits [12]. As the measles vaccine mandate came into effect at about the same time as the COVID-19 pandemic reached Germany, the pandemic is to be considered as an important context factor for the implementation of the mandate.

Because the measles vaccine mandate is a novelty in Germany, there are no scientific studies to date that examine its implementation in general or the role of the LHDs in particular. Therefore, this study aimed (i) to examine how the mandate was implemented, (ii) to identify operational challenges encountered during its implementation, (iii) to assess how the COVID-19 pandemic shaped the implementation, and (iv) to investigate public health expert attitudes towards the measles vaccine mandate. Through this investigation, the study endeavors to provide critical insights into the mandate’s role in the broader context of public health policy and vaccine uptake strategies. By elucidating key factors affecting the mandate’s execution, this research provides evidence-based recommendations aimed at improving the implementation and effectiveness of future vaccination policies.

The focus of this study is solely on the new requirement to provide proof of measles immunity. Other regulations described in the mandate, such as extended reporting requirements for other infectious diseases [2], were not considered.

## Methods

A qualitative study design was chosen to gather detailed and differentiated descriptions of experts’ experiences and opinions, allowing for a comprehensive understanding of the stated research interest.

### Study setting

This study focused on the public health landscape in Germany, a politically stable, high-income country located in Central Europe. In Germany, the responsibility for public health planning and decision-making largely falls to the federal states. These subnational entities possess their own governments and parliaments, allowing for regional autonomy in a wide range of policy areas while operating within the overarching national health framework.

In April and May 2021, about 14 months after the introduction of the mandate and the onset of the COVID-19 pandemic in Germany, we conducted online interviews with employees from different LHDs to explore their opinions on and experiences with the implementation of the measles vaccine mandate. While one interview involved two employees from the same LHD, all other interviews were conducted as one-on-one meetings.

### Study participants and recruitment

**Fig 1:**
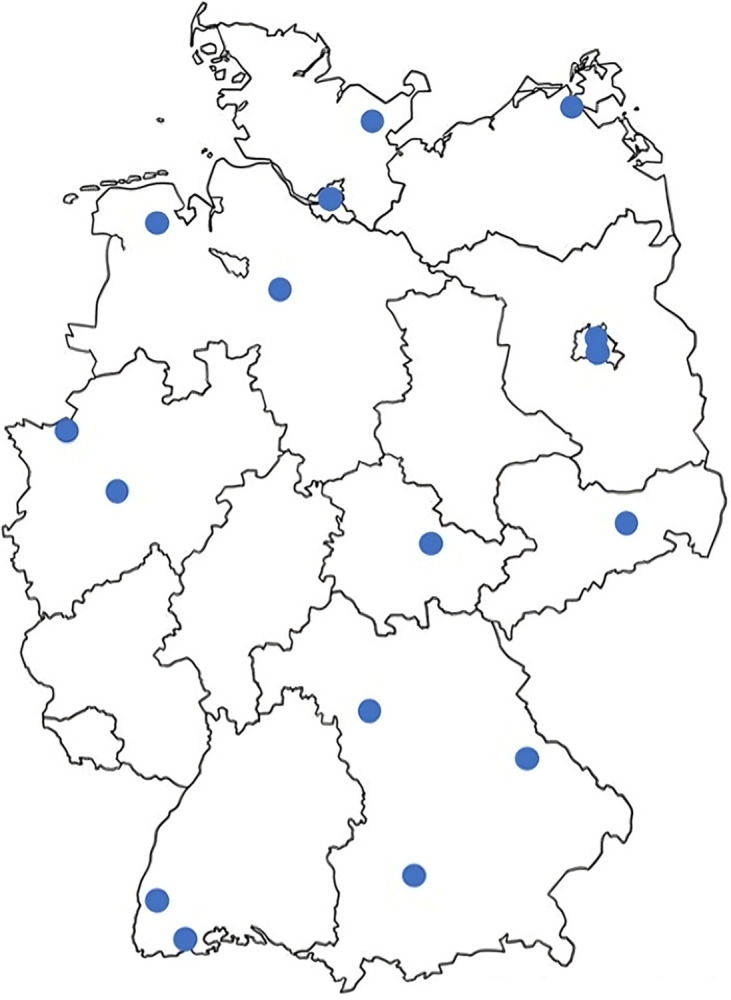
Localization of the 16 participating local health departments in Germany.

The study sample consisted of employees from German LHDs who were directly involved in the implementation of the measles vaccine mandate, herein referred to as experts due to their specialized knowledge and pertinent experiences [13]. All participants were required to be at least 18 years old. To assemble the study sample, purposive sampling was employed. Potential participants were approached during two separate public health conferences related to vaccination efforts in March and April 2021, where the planned study was presented and participation was encouraged. Interested individuals were invited to contact the research team via a study-specific email address. The final sample included 17 experts representing 16 different LHDs across 10 German federal states (Fig 1).

### Data collection

Data were collected within online semi-structured interviews via *WebEx*, guided by an interview protocol consisting of open-ended questions (S1 Appendix). Semi-structured interviews are based on a catalog of questions, which, however, can be flexibly adapted to the interviewee and the flow of the interview to investigate the research interest in greater depth based on the responses given [14]. Accordingly, a modification of the order of topics or additional follow-up questions were possible [14]. The interview guide was developed by a team of researchers with expertise in immunization programs and evaluation research. The following topics were covered by the guide: (1) planning and preparation of the mandate, (2) implementation of the mandate (proof of immunity), (3) communication and counseling, (4) parental acceptance of the mandate, (5) implementation of the mandate (sanctions), (6) impact of the COVID-19 pandemic, and (7) strengths and weaknesses of the mandate. The development of the interview guide followed the SPSS principle (collect, check, sort, and subsume) by Helfferich [15]. The instrument was pretested within the study team and with two individuals from the target population to validate its logic and comprehensibility. Study participants were provided with a summary of the main topics prior to the interviews.

The interviews were conducted by the first author (SW, female, trained and experienced in qualitative research methods), who had no contact with any of the interviewees prior to this study. All interviews were conducted in German, audio-recorded using an encrypted voice recorder, and transcribed verbatim following the basic transcription system of Dresing and Pehl [16]. Interview lengths varied from 30 to 90 minutes. During transcription, any information that could potentially allow a direct link to an individual was anonymized. Participants could request a transcript of their interview, fostering transparency and participant validation of the data collected.

### Data analysis

Data analysis was conducted using the Framework method, a specific approach to qualitative content analysis, to systematically manage and analyze the qualitative data [17]. The thematic categories (codes) were deductively derived from the structure and content of the interview guide. A coding tree, which organizes codes hierarchically, facilitated the systematic application of the established codes to the interview transcripts, enabling the identification of relevant text segments. All interview transcript excerpts relevant to our research interest were generalized and summarized in a stepwise process. The aggregated study results on the main themes and associated sub-themes were organized in a matrix with the dimensions “cases x categories*”*. This organization allowed for the comparison and interpretation of data across cases as well as across categories within a single case [17]. To ensure consistency, the interviewer (SW) coded and analyzed the data. Data management and analysis were facilitated by the software *MAXQDA*.

### Ethics

Participation was voluntary. All study participants received detailed study information by mail prior to data collection, which outlined the study’s objectives, procedures, ethical considerations, including confidentiality measures, and emphasized the voluntary nature of participation. The interviews were based on written, informed consent. There was no compensation for study participation.

The study’s protocol and related documents received approval from the data protection officer of the Robert Koch Institute, ensuring adherence to data protection standards. Given that this study did not involve the collection or analysis of sensitive personal data beyond the professional role, the research project did not require the oversight of an ethics committee according to German regulations. This exemption applies as this study did not engage with areas mandatorily subject to ethics committee approval in Germany, such as studies involving drugs, biologics, medical devices, clinical trials, the use of human biological materials, genetic research, or studies on embryos, stem cells, and cloning, as stipulated by the Drug Law, the Medicinal Products Law, and the Medical Device Law Implementation Act, among others. We confirm that all research methods were conducted in accordance with the relevant guidelines and regulations, ensuring ethical integrity. The reporting of this study was guided by the Consolidated Criteria for Reporting Qualitative Research: 32-item checklist [18].

## Results

The experts in this study worked as physicians (n = 11, 64.7%), specialists in social medicine (n = 3, 17.6%), or administrators (n = 3, 17.6%) and were responsible for implementing the measles vaccine mandate in their LHD. Most of the interviewees were female (n = 15, 88.2%). A large proportion (n = 11, 64.7%) was active in the pediatric and adolescent medical service. The characteristics of the study sample are summarized in Table 1.

**Table 1:** Characteristics of the study participants (N = 17).

Characteristics	Total sample n (%)
**Gender**	
** Male**	2 (11.8)
** Female**	15 (88.2)
**Profession**	
** Physician**	11 (64.7)
** Specialist in social medicine**	3 (17.6)
** Administrator**	3 (17.6)
**Affiliated department in the local health department**	
** Pediatric and adolescent medical service**	11 (64.7)
** Medical service**	3 (17.6)
** Hygiene and environmental medicine**	1 (5.9)
** Health protection**	1 (5.9)
** Administration**	1 (5.9)

### Implementation of the measles vaccine mandate

This section provides a summary of how the experts described the implementation process of the measles vaccine mandate. Typically, the proof of immunity is initially checked in the relevant facilities, such as schools, preschool childcare centers, and healthcare facilities. The results are documented and the individuals who did not provide immunity evidence are reported to the responsible LHD. In instances of ambiguous vaccination cards, medical certificates (e.g., for contraindications) or laboratory reports (e.g., for antibody verification), the facilities are often supported by the LHD in the verification process. LHDs are responsible for documenting and addressing reports of absent immunity proofs from institutions. Most interviewees indicated that they do not have a complete overview of the extent of missing proofs. Many institutions had not yet reported to the LHDs at the point of data collection, possibly due to the transition period, leading experts to speculate that several institutions had not yet initiated immunity proof verification. Conversely, some facilities had completed this process and informed the LHDs of any missing proofs. Some study participants speculated that the extent to which immunity was already being checked depends on the facility management’s stance on the mandate and its perceived significance.

As of the data collection period, about two-thirds of interviewees were engaged in addressing instances of missing immunity proofs, adopting a phased approach. Initially, LHDs remind the individuals in question of their obligation via phone or mail. Following this, multiple reminders are usually sent, sometimes specifying deadlines and hinting at possible sanctions. A few experts mentioned offering personal consultations. Other LHDs did not provide in-person counseling due to the pandemic or a lack of personnel resources.

Most participants had not yet enforced sanctions at the time of data collection, which become relevant if immunity proofs remain unsubmitted despite several reminders. The approach to processing the reports and enforcing sanctions varied across LHDs. Several study participants noted that sanctions are typically enacted by an authority other than the LHDs, such as the regulatory office. In some LHDs, it was still unclear how to apply sanctions, with unresolved questions about, for example, the amount of the fine. Other interviewees indicated values between EUR 1,000 and 2,500, noting differences between different groups of people or increased fines for repeated requests.

For most study participants, limited time was available to implement the measles vaccine mandate, for example due to the pandemic. However, for other experts, activities related to the mandate took up a large part of their work within the LHD. The amount of work associated with the mandate was observed to fluctuate over time and was expected to increase significantly after the transition period ends and individuals who were either employed or under care before March 2020 will be required to provide proof of their immunity.

### Challenges in the implementation of the measles vaccine mandate

Many experts pointed to the prevalent uncertainty around the mandate’s implementation procedures as a significant challenge. This uncertainty encompassed issues such as handling fraudulent medical certificates and determining the course of action for imposing sanctions, including defining responsibilities and setting the amount of fines. Such ambiguity led to considerable differences in how different LHDs executed the mandate. Many interviewees felt left alone with these questions and criticized the lack of designated contacts and support from higher-level institutions.

„The main problem […] is that the support for these problems that arise in everyday life is basically not there or is much too slow. In other words, there is no contact person who can really explain in a timely manner: this is how we can deal with it. Or that there is simply far too little feedback—How should we implement it? How should we deal with these problem cases […]?" (Participant 5, physician, female)

The interviewees reported that the prevailing legal framework allows every LHD to adopt a different approach to the implementation of the measles vaccine mandate. Consequently, many study participants advocated for the establishment of standardized procedural instructions or guidelines for the whole country or at least within each federal state. It was pointed out several times that considerable regional differences in the implementation of a national policy are not appropriate and could lead to lower acceptance by the public.

The capability of facilities to verify immunity and report absent proofs was reportedly limited by the fact that non-medical personnel may lack the expertise to scrutinize vaccination cards and medical certificates adequately. Reportedly, this limitation resulted in the acceptance of potentially fraudulent documents. Fraudulent certificates are incorrect medical certificates (often vaccination or test certificates for communicable diseases) issued by a medical professional. Dealing with this type of medical certificate was described by almost all interviewees as a major challenge.

„In the neighboring county, there was a doctor working in a private practice […] They have issued umpteen certificates, both for the mask exemption and of course for the exemption from the vaccination obligation. That has been quite lucrative: a certificate costs 300 euros there. […] People traveled from the Allgäu and from Thuringia and from hundreds of kilometers away […] This certificate is formulated so that they certify not only an individual contraindication against a measles vaccination, but the formulation that this person may not be vaccinated at all with any vaccine now and in the future.” (Participant 7, physician, male)

Some experts noted that they were internally ordered not to doubt apparent fraudulent certificates principally. Other interviewees reported fraudulent documents or the physician who issued the fraudulent certificates to the state medical association. In this context, it was often noted that there would always be groups of people who will not be persuaded to get vaccinated and will evade their obligation to provide proof.

„And the opponents of vaccination, to be honest, they cannot be caught by any mandate in this world. That is my personal conviction. So, that one assumed: now all [parents] have their children vaccinated—this is not going to happen.”(Participant 12, specialist in social medicine, female)

Several experts suspected that the actual extent of vaccine-critical individuals would become apparent after the transition period has expired, as some individuals refer to this deadline and refuse to submit the proof earlier.

The participating experts reported few challenges in the interaction with parents of children attending school or preschool childcare as one of the largest groups affected by the mandate. However, they identified different levels of difficulty depending on whether the child had already been vaccinated, had simply not been vaccinated because the parents had forgotten, or whether the parents were opposed to vaccination. Parents of vaccinated children, and thus the majority, did not seem to have any problems with the mandate.

„The vast majority [of parents is] uncritical, because the majority is vaccinated. […] Those who have vaccinated their children do not find it dramatic, because they have to show the vaccination card anyway, for example at the school entry examination. So it is nothing out of the ordinary for parents who have already taken children through such a procedure at some point. Many also think it is good that it is now mandatory.”(Participant 12, specialist in social medicine, female)

Parents who have simply forgotten their obligation to provide proof usually complied after the first request. LHDs also had to deal with people with a vaccine-critical attitude, who feel that their fundamental rights are being restricted, regard vaccination as bodily harm, engage in lengthy discussions or threaten legal action. However, extreme negative parental reactions were described to be rare.

„I would say [that vaccine-critical attitudes are] fortunately not common. But when [they are vaccine opponents], they are real [opponents]. So these are parents who make it very clear to us on the phone, in the first meeting what they think of the whole issue. At times, discussions extend beyond factual matters into personal territory. So I also had to end a phone call […] because it was no longer on the same level, the conversation with the mother.”(Participant 11, specialist in social medicine, female)

### Impact of the COVID-19 pandemic on the implementation of the measles vaccine mandate

According to most experts, the impact of the COVID-19 pandemic on the implementation of the measles vaccine mandate was substantial. Infection control activities often occupied the majority of the working hours in the LHDs. As a result, regular activities, including the implementation of the mandate, were often put on hold or, in some cases, completely suspended for certain periods. Since the COVID-19 pandemic reached Germany at about the same time as the mandate came into effect (in March 2020), meetings, working groups, and preparations that had been planned for the mandate were often canceled. According to the experts, the implementation of the mandate was immediately displaced by the pandemic in both public awareness and prioritization in the LHDs.

Some experts feared that the pandemic could also have a negative impact on vaccination rates for other infectious diseases (such as measles), further undermining the intention of the mandate. One of the reasons given was that doctor visits were less frequent and, accordingly, the vaccination status for infectious diseases besides COVID-19 was less likely to be checked and completed. Furthermore, parents were sometimes unable to provide proof due to COVID-19-related infection control measures, such as school closings. These circumstances often delayed the check for immunity.

A few interviewees reported that their work was not or only slightly affected by the pandemic, mostly because they were not assigned or only partially assigned to COVID-19-related activities. Positive effects of the pandemic were rarely mentioned, including an increase in public support for the measles vaccine mandate and a shift in attitudes towards vaccinations in general.

„It [*the mandate*] has experienced a higher level of acceptance. I am convinced that the danger or the value of infectious diseases has now come into consideration again for the first time in this generation. […] I believe that this actually means a rethinking in the direction of vaccination in general as well. People will think much more about: how can I avoid diseases? Perhaps, I hope in any case, this will also have an effect on the existing vaccinations, so that the willingness to vaccinate will at least remain at the same level or even increase.”(Participant 16, physician, female)

### Experts’ attitudes towards the measles vaccine mandate

Most experts suspected that the measles vaccination rate would be increased through the mandate and that some vaccination gaps would be closed. Some experts even suggested that the new legislation is the only way to increase measles vaccination rates. It has been noted that it has brought more attention to measles and vaccinations in general, and has highlighted their relevance.

„Basically, I think it is a nice thing to show people that this is really important. […] Sometimes it is also easier if you are not given a choice, but are simply told: do this, do that.” (Participant 4, physician, female)

Some interviewees reported their work becoming easier due to the mandate, since the legal basis clearly regulates the obligations of the parties involved. Furthermore, the legislation emphasizes the social responsibility of citizens in preventing infectious diseases.

„But overall I think it [*the mandate*] is good. Because I think that at the moment we are developing a bit socially in a direction where everyone knows their rights, but the duties that you also have in a community are being neglected a bit.”(Participant 14, physician, female)

However, others were more hesitant, as they saw alternative approaches or necessary supplements to the mandate, such as more intensive public relations work, reporting, education, and regular, easily accessible vaccination campaigns.

„I would rather like to put the energy I am putting in here now into a vaccination campaign or into a medical activity in schools with vaccination counseling […]. With the same effort, I could visit every school in our county over the year and do at least one vaccination consultation with simultaneous vaccination of those who are in default [with their vaccination]. […] Then this would actually be an action and not just an administrative activity.” (Participant 16, physician, female)

The experts also expressed their opinions on the imposition of sanctions in the context of the mandate. Almost all participants considered sanctions appropriate and effective. They mentioned that penalties are necessary to make people act and to give the mandate a certain importance. In particular, people who have forgotten about vaccination or have not yet taken care of it were reached by the sanctions and reminded with pressure.

„I have now learned in the process that with certain people who, as I said, need […] [a bit pressure], I would say that fines make sense. Something only happens when there is pressure. And I would also take advantage of that. That has to happen, because it is about the well-being of the child. So you can push the parents in the right direction and that can also cost [them] something.”(Participant 16, physician, female)

In some cases, experts pointed out that individuals with well-founded fears, possibly stemming from negative experiences with vaccinations, might be reluctant to vaccinate their children. For these people, the imposition of sanctions was considered problematic. Some experts were ambivalent about the sanctions.

„I personally think that sanctions do not really reach people, on one hand. But on the other hand, you kind of need a plan so that it is taken seriously at all. And also with regard to people who make the effort and who stick to it and who participate. It would be unfortunate if they get the impression: we stick to it, we do everything / or even the facility managers: we make the effort and check it, and if you do not care, then it does not matter. […] And in order to attach a high value to it [*the mandate*], it is not possible without imposing sanctions.” (Participant 8, physician, female)

Other interviewees were fundamentally critical of sanctions and doubted that people would be persuaded to vaccinate because of a fine. They would prefer to rely on enhanced public relations efforts and to increase vaccination willingness through more extensive education and counseling. There was also frequent criticism that wealthier parents of school-aged children can buy their way out of the obligation because of the priority given to compulsory schooling, and that financial penalties in this context are ineffective.

## Discussion

With this study, we contribute to the evaluation of the new measles vaccine mandate in Germany, more specifically of its implementation and associated challenges. Data were collected in April and May 2021, about 14 months after the introduction of the mandate and the start of the COVID-19 pandemic in Germany. Our results show that the implementation of the measles vaccine mandate was often delayed and heterogeneous across LHDs. The main challenge in implementing the mandate was the prevalent uncertainty about the implementation procedure itself, resulting from the lack of procedural guidance. As an important contextual factor, the COVID-19 pandemic usually led to a substantial delay in implementation.

The status and process of the mandate’s implementation varied considerably across both facilities and LHDs. While some facilities had already checked and reported all missing proofs of immunity, others had not yet contacted the responsible LHD. While some LHDs had not yet contacted individuals reported, others were already following up on reports. In doing so, they followed different procedures. The mandate was not implemented with the same priority and speed in different LHDs during the first year after it came into effect. It is to be expected that the implementation process will take some time, especially during major public health events such as the COVID-19 pandemic. A follow-up study should evaluate if the mandate is ultimately being implemented by all facilities and LHDs as intended or whether blind spots remain.

At the time of data collection, few LHDs had already imposed sanctions. This was partly because implementation of the mandate had been delayed by the COVID-19 pandemic. This situation also reflects the transition period granted for the implementation of the mandate, which states that individuals already employed or cared for in the facilities as of March 1, 2020 officially did not have to expect sanctions until the transition period expires. At the time of data collection, the transition period ended in July 2021, but was later extended to July 2022 [11]. However, some LHDs have decided to follow up on missing proofs of immunity before the end of the transition period. In some cases, this led to conflicts with individuals who insisted on complying with the transition period and refused to provide the proof beforehand. Therefore, some experts’ assumption that the actual extent of vaccine-critical individuals would not become apparent until after the transition period has expired is plausible. Further research should assess whether this will turn out to be true.

Interviewees highlighted a range of challenges associated with the implementation of the mandate. The main challenge was dealing with the uncertainty about the implementation procedure itself. LHDs found themselves needing to develop their own approach and criticized the resulting procedural heterogeneity among LHDs. Forman et al. [19] noted that divergent vaccination policies and enforcement procedures could diminish public trust and fuel vaccine hesitancy. Although this consideration originally related to national vaccination policies involving AstraZeneca’s COVID-19 vaccine, the underlying concerns are applicable to our context as well. Many experts wished for uniform procedural guidelines at least at the federal state level. Indeed, the German Infection Protection Act outlines the basic requirements and responsibilities for implementing the mandate, but it does not specify how to proceed in fulfilling these duties. The European Observatory on Health Systems and Policies clearly highlights the value of a harmonized approach to the organization and delivery of vaccination services [20]. Providing clear procedural guidance early on could have helped LHDs, saved resources, and potentially contributed to greater public acceptance of the mandate.

Another challenge was handling fraudulent medical certificates, which often do not contain medical diagnoses and are sometimes marketed for profit. Professional medical regulations provide some support to LHDs in this regard, as medical certificates without confirmed medical diagnoses are not considered to be valid [21]. Furthermore, physicians who issue incorrect medical certificates put themselves at risk of imprisonment or fines [22]. While some experts were ordered not to doubt such documents, others proactively followed up on them and reported the certificate or the certifying physician to the state medical association. LHDs expressed a need for support from higher-level institutions, such as clear guidance on how to proceed in such cases. Policy-makers should anticipate the occurrence of fraudulent certificates and help LHDs deal with them appropriately.

In this context, dealing with vaccination opponents is a common challenge for LHDs. Contact with the majority of individuals, who could not provide proof of immunity but were open to vaccination, went smoothly. However, vaccine-critical attitudes resulted in a high workload. Previous research estimated the proportion of parents with a (rather) opposing attitude towards vaccination in Germany at 7% [23]. In addition to vaccine-critical attitudes, there may be many other reasons why individuals are not vaccinated. A German survey study identified the most frequent reasons for lacking measles vaccination as not knowing about the need for vaccination (62%) and not being aware of belonging to a group to which vaccination is recommended (22%) [23]. Providing adequate information and public education would raise awareness and counteract the main reason for non-vaccination, namely, lack of knowledge.

With the strict enforcement of mandatory immunization, including financial penalties, social restrictions, and allowing only medical exemptions, the German measles vaccine mandate can be classified as a hard mandate according to the classification by MacDonald et al. of mandatory immunization programs [24]. Most interviewees were convinced of the mandate’s value and suggested that the additional checks and the immunity proof requirement would increase the measles vaccination rate. This expectation aligns with the results of previous research that has shown that mandatory vaccination and the linkage with financial penalties can improve vaccination coverage [25–27]. However, evidence also suggests only minimal differences in vaccination rates between contexts with vaccination recommendations and those with vaccination requirements [28]. According to Omer et al. [29], flexible vaccination policies, as opposed to coercive and punitive ones, may be advantageous.

Some experts also pointed out that strict opponents of vaccination would not be persuaded to vaccinate by the mandate. The regulations still offer too many ways to evade the obligation to provide proof and vaccination, such as fraudulent certificates, legal delay tactics, and the repeated payment of fines for school-aged children. Previous research has already recognized that individuals with strong objections to vaccination, despite a legal requirement, find loopholes to avoid the obligation to provide proof [30]. Overly strict mandates are considered to reinforce these phenomena and fuel anti-vaccine attitudes [29]. In this context, it is also important to consider that compulsory vaccination can exacerbate social inequities, as financial penalties disproportionately affect deprived groups [11,29]. Children of migrant parents, for example, have a 10% lower immunization rate for booster vaccinations in Germany [31], likely attributed to access barriers. Since poverty, social exclusion, and barriers to access can depress vaccination rates, it is important to ensure that vaccines are easily and safely accessible to all societal groups before introducing a legal requirement [24,29]. An alternative approach to financially penalizing the non-vaccination is providing financial incentives for vaccination. This approach helped Australia increase the percentage of children completely immunized from 84.3% in 1997 to 93.5% in 2000 [32].

Several interviewees were rather skeptical of the mandate, as they preferred alternative approaches or necessary additions to the mandate, such as more intensive public relations work, education, and easily accessible vaccination campaigns. The measles vaccine mandate was introduced at a time when policymakers felt there were few alternatives, as traditional and less coercive approaches had not sufficiently increased measles vaccination coverage [1]. Some experts seemed to see voluntary approaches as having greater leverage to increase vaccination rates. In contrast, Brewer et al. [33] suggested that attempts to change attitudes towards vaccination have rather little impact on vaccination rates. In general, mandatory vaccination should not be a stand-alone policy but part of a comprehensive package that includes public information, robust immunization recording, and reliable safety monitoring [29]. In addition, specific strategies to increase vaccination rates must always match the reasons why people do not get vaccinated [34].

Our results show that the COVID-19 pandemic was often related to a considerable delay in the implementation of the measles vaccine mandate and the cancellation of mandate-related preparations. According to most experts, the mandate quickly lost much of its public awareness and priority in the LHDs upon its entry into effect. Indeed, around the same time that the mandate came into effect, the COVID-19-related workload in LHDs increased exponentially [12]. Tasks like contact tracing, managing quarantine orders, and testing for COVID-19 successively dominated their daily workload [12]. This underlines that the context for the introduction of the measles vaccine mandate was challenging. In general, making vaccination a legal requirement must be done with consideration of the context. This includes considering both temporal factors, like the pandemic, and underlying factors, such as societal composition and social and health inequities [11,24,28,29].

### Limitations

This study examines the implementation of a new German vaccine mandate to strengthen measles prevention, provides new insights into the operational work of the public health service, and highlights the need to harmonize the implementation of health policy measures among executing entities.

However, some limitations need to be considered. The interviewees were asked about past situations and activities. Retrospective assessment is inherently associated with difficulties because interviewees may not or may only partially remember all events correctly [35]. Memory gaps or false memories are difficult to assess or recognize by outsiders, but the potential for this bias must be taken into account. In general, using self-generated samples carries a risk of selection bias. Individuals who have agreed to participate in the study may systematically differ from non-participants, for example, in their attitude toward the measles vaccine mandate. The first author (SW) solely analyzed the data, including category construction and coding, and the coding process was conducted once. Since only one coder was involved, intercoder reliability does not apply. The study did not assess intracoder reliability, potentially affecting the consistency of coding over time. However, since the analysis was less interpretative than summarizing and structuring the interviewees’ statements, we suggest that this limitation does not significantly diminish the results’ conclusiveness.

## Conclusions

Our study provides a comprehensive evaluation of the implementation of the measles vaccine mandate in Germany and the operational challenges associated with it one year after the mandate came into effect. The different implementation status and approaches among LHDs underscore the complexities involved in enforcing such mandates, from dealing with procedural uncertainties to addressing fraudulent medical certificates and vaccine hesitancy. A harmonized approach would likely have led to more certainty in the implementation, resource savings in LHDs, and higher public acceptance of the mandate. Despite the belief in the mandate’s potential to improve measles vaccination rates, our findings highlight the necessity of a multifaceted strategy that goes beyond legal requirements. This includes public education, awareness campaigns, accessible vaccination programs, and addressing societal inequities that may hinder vaccine uptake. The experience with the measles vaccine mandate, set against the background of a global health crisis, emphasizes the need for flexible, context-aware policies that can adapt to changing health landscapes and societal needs. Future efforts should focus on bridging the gaps in mandate implementation, ensuring equitable access to vaccines, and fostering public trust and compliance through transparent, inclusive public health strategies.

## Supporting information

S1 Appendix Interview guide for collecting qualitative data in our study. (PDF)

## Abbreviations

COVID-19: Coronavirus disease 2019
LHD: Local Health Department
STIKO: National Immunization Technical Advisory Group in Germany
