# Supplementary material for: The implementation of a new measles vaccine mandate in Germany: A qualitative study in local health departments
Source: PLoS One. 2024 Jun 25;19(6):e0306003. doi: 10.1371/journal.pone.0306003 (PMC11198778; doi:10.1371/journal.pone.0306003)
Supplement: S1 Appendix — (PDF) [file pone.0306003.s001.pdf]

S1 Appendix. Interview guide for collecting qualitative data in our study.

First, I would like to ask about your current professional activities and your role in the implementation of the measles vaccine mandate *(MSG)*.

- What is your current job title at the local health department?
- Which department do you belong to?
- What is your role in the local health department and what are your core responsibilities?
  - *How long have you been involved in the implementation of the MSG?*
  - *Please describe your responsibilities and duties since you began implementing the MSG.*
  - *What percentage of your total workload is MSG-related?*
- How many employees in your local health department are also involved in implementing the measles mandate?
  - *What tasks do these people take on?*

1. Planning and preparation of the mandate

Now I would like to talk with you about the initial phase around the introduction of the MSG.

1.1. When and how did you hear about the MSG regulation?

1.2. Please describe the organization of the initial phase around the introduction of the MSG in your local health department.

- *How was the interaction with other stakeholders involved in the introduction of the MSG? (e.g., ministries of health, education)*
- *To what extent were representatives of schools and preschool childcare as well as pediatricians or general practitioners involved?*

1.3. How has your local health department prepared for the MSG?

- *To what extent additional resources were made available for the implementation of the MSG? (e.g., additional personnel, information material)*
- *How did you communicate your new responsibilities to the community?*
- *How were you personally prepared for the new tasks?*
  - o *What would you have wished for in addition? (e.g., further training)*

2. Implementation of the mandate (proof of immunity)

An important part of the MSG is controlling the proof of immunity (e.g., via the vaccination card/medical certificate). The regulations differentiate between the individuals who entered a concerned facility for the first time after 01.03.2020 and the individuals who were already working or being cared for in the facilities as of March 1, 2020. For the second group, the transition period applies until the end of 2021.

2.1. How is measles protection checked in general?

- *Are vaccination cards checked by the facilities in accordance with legal regulations?*

- *How are the inspections documented?*

2.2. Please describe to what extent the proofs of the individuals affected by the transition period are already checked.

2.3. What experiences do you have so far in the cooperation with facilities?

3. Communication and counseling

The MSG affects different groups of people, and they certainly have different needs for advice and different questions.

How do you proceed in communicating and advising these people?

4. Parental acceptance of the mandate

One topic that particularly interests me is parental acceptance. I would like to hear more about the reactions of parents of children who are subject to the obligation to provide immunity proof.

4.1. How do parents generally react to their children's need for proof?

4.2. Please describe your experience with parents who <u>cannot</u> provide evidence of measles protection for their children.

4.3. How common is it that parents do not <u>want</u> to provide evidence of measles protection for their children?

- *Please describe your procedure in such a case.*
- *What strategies do you use to increase parental awareness of the need for immunization?*

5. Implementation of the mandate (sanctions)

The MSG also regulates the imposition of sanctions if someone does not comply with the obligation to provide proof. I would like to know more about your experiences in this regard.

5.1. What sanctions have you already imposed?

5.2. How do you proceed when imposing sanctions?

- *When is a sanction imposed?*
- *What is the time period for imposing sanctions and, if applicable, follow-up sanctions?*
- *How high are the fines usually?*

5.3. What is the procedure if measles protection is not proven or completed despite the imposition of a sanction?

- *How often does this happen?*

5.4. What happens if a person cannot pay the fines due to financial hardship?

5.5. How do you personally feel about such sanctions?

6. Impact of the COVID-19 pandemic

The COVID-19 pandemic has more or less turned all our lives upside down. Local health departments in particular are heavily involved in infection control measures. I would now like to learn more about the impact of the pandemic on the implementation of the MSG.

To what extent has the COVID-19 pandemic and related measures impacted the implementation of the MSG?

- *How did you perceive that - to what extent did the pandemic affect the awareness or visibility of the MSG?*
- *To what extent have tasks related to the MSG been deprioritized?*
- *What was the impact of preschool childcare/school closures and home office work on the implementation of the MSG?*

7. Strengths and weaknesses of the mandate

The primary objective of the mandate is to close immunization gaps and increase vaccination rates.

7.1. What do you think - to what extent can the MSG achieve this overall objective?

- *What do you think are the strengths of the MSG?*

7.2. With regard to which aspects do you think the MSG does not meet the expectations/objectives?

- *What do you think are the weaknesses of the MSG?*
- *What approaches can you suggest to address the associated problems/weaknesses of the MSG?*

7.3. What alternatives can you think of to increase measles vaccination rates?

7.4. The MSG has also resulted in new tasks for the local health departments. Among other things, you are now responsible for implementing the mandate at the local level. Has this new role changed your relationship with citizens?

- *What do you think about this new role?*

Is there anything else you would like to mention with regard to the MSG?
